# Supplementary material for: A fault-intrusion-tolerant system and deadline-aware algorithm for scheduling scientific workflow in the cloud
Source: PeerJ Comput Sci. 2021 Nov 2;7:e747. doi: 10.7717/peerj-cs.747 (PMC8576554; doi:10.7717/peerj-cs.747)
Supplement: Supplemental Information 6 [file peerj-cs-07-747-s006.zip › Myproject1.1/docs/org/cloudbus/cloudsim/core/class-use/SimEntity.html]

Uses of Class org.cloudbus.cloudsim.core.SimEntity


JavaScript is disabled on your browser.


- Overview
- Package
- Class
- Use
- Tree
- Deprecated
- Index
- Help

- Prev
- Next

- Frames
- No Frames

- All Classes

## Uses of Class org.cloudbus.cloudsim.core.SimEntity

- Packages that use SimEntity

  | Package | Description |
  | --- | --- |
  |  |  |
  | --- | --- |
  | org.cloudbus.cloudsim |  |
  | org.cloudbus.cloudsim.core |  |
  | org.cloudbus.cloudsim.examples |  |
  | org.cloudbus.cloudsim.network.datacenter |  |
  | org.cloudbus.cloudsim.power |  |
  | org.workflowsim |  |
- - ### Uses of SimEntity in org.cloudbus.cloudsim

    Subclasses of SimEntity in org.cloudbus.cloudsim

    | Modifier and Type | Class and Description |
    | --- | --- |
    |  |  |
    | --- | --- |
    | `class` | `Datacenter` Datacenter class is a CloudResource whose hostList are virtualized. |
    | `class` | `DatacenterBroker` DatacentreBroker represents a broker acting on behalf of a user. |
  - ### Uses of SimEntity in org.cloudbus.cloudsim.core

    Subclasses of SimEntity in org.cloudbus.cloudsim.core

    | Modifier and Type | Class and Description |
    | --- | --- |
    |  |  |
    | --- | --- |
    | `class` | `CloudInformationService` A Cloud Information Service (CIS) is an entity that provides cloud resource registration, indexing and discovery services. |
    | `class` | `CloudSimShutdown` CloudimShutdown waits for termination of all CloudSim user entities to determine the end of simulation. |

    Methods in org.cloudbus.cloudsim.core that return SimEntity

    | Modifier and Type | Method and Description |
    | --- | --- |
    |  |  |
    | --- | --- |
    | `static SimEntity` | CloudSim.`getEntity(int id)` Get the entity with a given id. |
    | `static SimEntity` | CloudSim.`getEntity(java.lang.String name)` Get the entity with a given name. |

    Methods in org.cloudbus.cloudsim.core that return types with arguments of type SimEntity

    | Modifier and Type | Method and Description |
    | --- | --- |
    |  |  |
    | --- | --- |
    | `static java.util.List<SimEntity>` | CloudSim.`getEntityList()` Returns a list of entities created for the simulation. |

    Methods in org.cloudbus.cloudsim.core with parameters of type SimEntity

    | Modifier and Type | Method and Description |
    | --- | --- |
    |  |  |
    | --- | --- |
    | `static void` | CloudSim.`addEntity(SimEntity e)` Add a new entity to the simulation. |
    | `protected static void` | CloudSim.`addEntityDynamically(SimEntity e)` Internal method used to add a new entity to the simulation when the simulation is running. |
  - ### Uses of SimEntity in org.cloudbus.cloudsim.examples

    Subclasses of SimEntity in org.cloudbus.cloudsim.examples

    | Modifier and Type | Class and Description |
    | --- | --- |
    |  |  |
    | --- | --- |
    | `static class` | `CloudSimExample8.GlobalBroker` |
  - ### Uses of SimEntity in org.cloudbus.cloudsim.network.datacenter

    Subclasses of SimEntity in org.cloudbus.cloudsim.network.datacenter

    | Modifier and Type | Class and Description |
    | --- | --- |
    |  |  |
    | --- | --- |
    | `class` | `AggregateSwitch` This class allows to simulate aggregate switch for Datacenter network. |
    | `class` | `EdgeSwitch` This class allows to simulate Edge switch for Datacenter network. |
    | `class` | `NetDatacenterBroker` NetDatacentreBroker represents a broker acting on behalf of Datacenter provider. |
    | `class` | `NetworkDatacenter` NetworkDatacenter class is a Datacenter whose hostList are virtualized and networked. |
    | `class` | `RootSwitch` This class allows to simulate Root switch which connects Datacenter to external network. |
    | `class` | `Switch` |
  - ### Uses of SimEntity in org.cloudbus.cloudsim.power

    Subclasses of SimEntity in org.cloudbus.cloudsim.power

    | Modifier and Type | Class and Description |
    | --- | --- |
    |  |  |
    | --- | --- |
    | `class` | `PowerDatacenter` PowerDatacenter is a class that enables simulation of power-aware data centers. |
    | `class` | `PowerDatacenterBroker` A broker for the power package. |
    | `class` | `PowerDatacenterNonPowerAware` PowerDatacenterNonPowerAware is a class that represents a non-power aware data center in the context of power-aware simulations. |
  - ### Uses of SimEntity in org.workflowsim

    Subclasses of SimEntity in org.workflowsim

    | Modifier and Type | Class and Description |
    | --- | --- |
    |  |  |
    | --- | --- |
    | `class` | `ClusteringEngine` ClusteringEngine is an optional component of WorkflowSim and it merges tasks into jobs |
    | `class` | `DatacenterExtended` DatacenterExtended extends Datacenter so as we can use CondorVM and other components |
    | `class` | `WorkflowEngine` WorkflowEngine represents a engine acting on behalf of a user. |
    | `class` | `WorkflowPlanner` WorkflowPlanner supports dynamic planning. |
    | `class` | `WorkflowScheduler` WorkflowScheduler represents a scheduler acting on behalf of a user. |

- Overview
- Package
- Class
- Use
- Tree
- Deprecated
- Index
- Help

- Prev
- Next

- Frames
- No Frames

- All Classes
